# Devon and Exeter Medical Society

**Published:** 1899-06

**Authors:** R. V. Solly

**Affiliations:** Hon. Sec.


### Devon and Exeter Medico-Chirurgical Society.

*November* 18*th*, 1898.

Dr. P. M. DEAS, President, in the Chair.

The PRESIDENT read a paper on "Sleep-producers and Sedatives." He called attention to the large number of drugs that were on the market, and to the fact that every doctor had his favourite one. Often the drug acted in the way of hypnotic suggestion. The doctor told the patient that he was go ng to give him something that would send him to sleep, and consequently the patient slept. The subject of the treatment of insomnia was most important, as the condition was on the increase. The following varieties of insomnia were distinguished: (1) Psychical or emotional insomnia, due to grief, &c.; (2) insomnia in connection with bodily disorder, such as pain, fever, diseases of the heart or urinary system, eczema, with errors of diet, such as abuse of tea, coffee, or alcohol; (3) insomnia which precedes or follows insanity. These cases are apt not to get treatment sufficiently early. *Physiology of sleep.*—According to the latest theory, the cortical cells or "neurons," and their processes "dendrons," are not in organic connection with the nerves which bring up afferent stimuli and carry away efferent orders. This is important with regard to the views held as to sleep, dreaming, and somnambulism. The essence of sleep is that the neurons are out of action. The telephonic exchange ceases to exist during sleep, and the lower nerve centres in the brain and spinal cord are depressed, together with a general lowering of the vital processes. As to the proximate cause of sleep, there are various theories; namely, that it depends on periodicity, fatigue of brain cells, or increased supply of blood to the brain. The last is certainly not true. There is diminished supply and pressure of blood in the brain due to contraction of vessels. The inhibiting action of the highest centres is in abeyance. Dr. Deas believed that sleep was due to the absorption by neurons from the blood of some material circulating in the blood, and that when a certain amount has accumulated sleep is produced. The conditions which tend to insomnia are—1, excitement and overaction; 2, depression (*a*) central or mental, (*b*) peripheral or bodily. In these conditions there is a state of vascular excitement, and the neurons are over-stimulated. The circulation is kept at such a state of tension that the neurons cannot be withdrawn from the irritation of the nerves, and their inhibiting action is still maintained. A drug may act as a sedative, and produce sleep, by putting the body into a more favourable condition for sleep. Unfortunately, nearly all sedatives are heart depressants. A sleep-producer acts directly on the neurons. He then called attention to some of the drugs available. *Bromides* had a remarkable effect in diminishing reflex irritation. Bromide of sodium, gr. 20, with tincture of lupuline ʒ i., was a good combination (at bedtime). In more urgent cases bromides must be given several times a day. In sleeplessness in connection with a rapid neurotic heart, digitalis may be combined with bromides. Bromide of potassium is the most depressing, and so is useful in cases of sexual excitement (with liq. extract of salix nigra). It is very useful in acute mania. *Chloral.*—Dr. Deas did not recommend this drug strongly. Chloral is better as a sleep-producer than as a sedative. It is uncertain and very depressing; occasionally useful when combined with bromides. He laid stress on the importance of only giving sleep-producers at bedtime, and when the conditions are favourable for the production of sleep. *Trional* is a good sleep-producer, and there is very little risk in its use. Grains 25 may be given for 2—3 nights running, and then a smaller dose. *Sulphonal* wants very careful watching. It is a treacherous drug, and is not to be depended upon. It is a powerful sedative, and does best for violent cases of acute mania. It is very useful if given at regular intervals and with proper precautions. It tends to interfere with the secretions, and has a cumulative action. It paralyses the peristaltic action of the bowels. It has a very depressing effect on the nerve-centres, causing ataxia, &c. Aperients are necessary during its use. In conditions of excited circulation with weakness, causing insomnia, do not hesitate to give stimulants with the sedative. In conditions of depression, causing insomnia—such as grief, melancholia, stupor, hypochondriasis, where there are vague feelings of discomfort, and mental pain—the neurons are in a state of irritation, very different from the feeling of comfort and well-being which precedes natural sleep. Here insomnia is well marked, and the depression is much worse during the sleepless hours. *Paraldehyde* is an ideal sleep-producer; it first stimulates the neurons, and then natural sleep is produced with no depression. In cases of melancholia with stupor, paraldehyde has sometimes a remarkable effect. After taking the drug the patient wakes up and talks for about ten minutes, and then falls fast asleep. The dose of paraldehyde is ʒi.; in exceptional cases ʒiij. to ʒiv. may be given, with liquid extract of liquorice to disguise the unpleasant taste. Dr. Deas did not advise drugs in all cases of insomnia. In cases of acute excitement ordinary means may act as well as drugs. The want of sleep is not so important in excited cases as in depressed cases. It may be necessary to prevent struggling, which would otherwise exhaust the patient. In general paralysis some authorities keep their patients during the stage of excitement in a condition of sulphonal intoxication, and so steer them into the haven of dementia. He did not advise the use of morphia or opium for insomnia without pain. These are narcotics, and not sleep-producers. In giving sleep-producers, give a sufficient dose that you think will produce sleep, and only at bedtime, and diminish the dose afterwards. In giving sedatives, begin with a small dose and go on increasing it. It is often more necessary to stimulate than to depress. Sleep is wanted, and not a condition of unconsciousness.

R. V. Solly, *Hon. Sec.*

